# Implementation of the INTERGROWTH-21^st^ gestational dating and fetal and newborn growth standards in Nairobi, Kenya: women’s experiences with ultrasound and newborn assessment: Women’s experiences with ultrasound and newborn assessment in peri-urban Kenya

**DOI:** 10.1080/16549716.2020.1770967

**Published:** 2020-06-16

**Authors:** Rachel M. Jones, Linda Vesel, Grace Kimenju, Teresa Ogolla, Meghan Munson, Sarah Little, Sathyanath Rajasekharan, Mary Nell Wegner, Ana Langer, Nicholas Pearson

**Affiliations:** a Department of Research & Design, Jacaranda Health, Nairobi, Kenya; b Department of Global Health and Population, Harvard T.H. Chan School of Public Health, MA, USA

**Keywords:** Pregnancy, antenatal care, ultrasound, patient perceptions, newborn health, implementation, growth standards

## Abstract

**Background:**

In order to make further gains in preventing newborn deaths, effective interventions are needed. Ultrasounds and newborn anthropometry are proven interventions to identify preterm birth complications, the leading cause of newborn deaths. The INTERGROWTH-21^st^ global gestational dating and fetal and newborn growth standards prescribe optimal growth in any population. Jacaranda Health in Kenya was the first low-resource health facility to implement the standards and evaluate their feasibility and acceptability.

**Objective:**

To capture patients’ perceptions of ultrasound and newborn care before and during implementation of the INTERGROWTH-21^st^ standards.

**Methods:**

The study was conducted over two years before and during the introduction of the INTERGROWTH-21^st^ standards. Fifty pregnant and/or newly delivered women were selected for in-depth interviews and focus group discussions using convenience and purposive sampling. Interviews were conducted by research assistants using semi-structured guides once in the pre-implementation phase and twice in the implementation phase. Interviews were transcribed, double-coded by two independent researchers and thematically analyzed together. Demographic information was obtained from hospital records.

**Results:**

Patients reported being generally satisfied with ultrasound care when providers communicated effectively. Women reported a priority for ultrasound was that it allowed them to feel reassured. However, a clear need for better pre-screening information emerged consistently from patients. Women noted that factors facilitating their choosing to have an ultrasound included ensuring the well-being of the fetus and learning the sex. Barriers included wait times and financial constraints. Patients were generally satisfied with care using the newborn standards.

**Conclusions:**

As the INTERGROWTH-21^st^ standards are implemented worldwide, understanding ways to facilitate implementation is critical. Increased and standardized communication about ultrasound should be provided before the procedure to increase satisfaction and uptake. Considering patient perspectives when integrating new standards or guidelines into routine clinical care will inform effective strategies in care provision, thus improving maternal and newborn health and survival.

## Background

Despite progress made in reducing under-five mortality worldwide, significant gaps remain in order to achieve Sustainable Development Goal 3 of ending preventable newborn and child deaths by 2030 [1]. With 47% of global child deaths occuring in the newborn period (first 28 days), addressing newborn health and survival is essential [2]. The burden of neonatal deaths is particularly pronounced in sub-Saharan Africa, where 39% of global deaths occur [2]. Thirty-five percent of neonatal deaths are a result of complications, of which the majority are related to low birth weight (<2.5 kgs) caused by intrauterine growth restriction and/or prematurity [3–6].

There are a number of proven interventions to manage newborns with preterm birth complications, including referral to tertiary care facilities for advanced care. Tools to support the identification of preterm birth before and after delivery include accurate gestational dating, fetal growth monitoring, detection of high-risk maternal conditions, management of preterm labor, and newborn anthropometry to identify small for gestational age (SGA) newborns. However, standardization of tools is needed to detect risk factors for and complications of preterm birth and to identify fetuses and newborns requiring specialized care; thus helping healthcare providers to make evidence-based clinical decisions [7,8]. Early ultrasound (before 24 weeks gestation), is specifically cited by the World Health Organization as being beneficial in establishing gestational age, detecting fetal anomalies, reducing post-term inductions, and promoting women’s positive pregnancy experience [9,10].

Patients’ perceptions of the quality of care they receive are equally important to the care provided by health workers in increasing access to and uptake of essential interventions [11,12]. Positive patient perceptions of facility-based care drive service utilization, return rates for services, and healthy behavior uptake, thus contributing to improved health outcomes [13–18]. Therefore, soliciting feedback from patients regarding their experience of care should be a critical component of any evaluation of new or improved clinical procedures, particularly those that rely on elective care-seeking [19,20].

The INTERGROWTH-21^st^ gestational dating and fetal and newborn growth standards were developed after a five-year prospective study in eight geographically-defined, healthy, urban populations across the globe [21,22]. The standards are designed to be utilized during obstetric ultrasounds and newborn size at birth assesments. Unlike previous population-specific reference charts, the INTERGROWTH-21^st^ standards describe how healthy fetal and newborn growth *should* progress in any population, thus allowing clinicians to identify when growth deviates from the standard, adjust clinical care, and execute interventions in a timely manner [7]. Jacaranda Health, a private maternity hospital in Kenya, was the first health facility in a low resource setting to implement and evaluate the feasibility and acceptability of the INTERGROWTH-21^st^ gestational dating and fetal and newborn growth standards as part of routine clinical practice. The primary objectives of this research were to (1) examine the factors that facilitated and impeded patients’ satisfaction with the care received and (2) understand patients’ attitudes and satisfaction with ultrasounds and newborn size at birth assessment. This paper captures the results related to patients’ experiences and perceptions of ultrasounds and newborn assessment before and during the implementation of the INTERGROWTH-21^st^ standards.

## Methods

### Study design

The results shared in this paper are part of a larger study, which was conducted over two years (March 2016 – March 2018), before (12-month pre-implementation phase) and during (12-month implementation phase) the introduction of the INTERGROWTH-21^st^ standards [23]. Before implementation of the INTERGROWTH-21^st^ standards, ultrasounds were not performed routinely and were only administered by an ultrasonographer upon patient request or when clinically indicated. Particular attention was paid during the implementation phase to increase the uptake of ultrasounds, which was the mechanism for implementing the INTERGROWTH-21^st^ gestational dating and fetal growth standards. The study protocol specified that any pregnant woman attending an antenatal care (ANC) visit between eight and 26 weeks of gestation during weekday business hours would be offered a free gestational dating scan. Fetal growth monitoring scans continued to be offered as needed and were performed by an ultrasonographer. Initially, three nurse-midwives were trained and certified to conduct the gestational dating ultrasounds in addition to their regular ANC duties; they rotated through a duty roster each week. Six months into the implementation phase, this model was changed to address clinical flow obstacles. The new model resulted in one certified nurse-midwife conducting all gestational dating scans without responsibility for providing other components of ANC. All live births delivered at Jacaranda Health were assessed using the newborn size at birth standards, which included measurements of length, weight and head circumference within 24 hours of birth.

Full details of the study design and methods can be found in the protocol paper [23]. Provider experiences, uptake of the standards, and the association between the implementation of the standards and clinical decision-making outcomes are discussed in a separate paper [24].

### Study setting

This study was conducted at Jacaranda Health, an 18-bed hospital in peri-urban Nairobi, Kenya. Jacaranda Health aims to provide high-quality, affordable care to low- and middle-income Kenyan women and their families. It runs on a nurse-midwife led model and provides ANC, vaginal and Cesarean delivery care, postnatal care, child wellness/immunizations, and family planning services. Women pay out-of-pocket and/or utilize private insurance schemes in order to seek antenatal and delivery services at Jacaranda Health. The costs of services for antenatal and delivery care are similar to those of other private hospitals in the area. However, the quality of services was rated as higher than similar private hospitals in the area by SafeCare, which measures quality standards for private hospitals in Africa [25].

#### Study participants

Study participants included patients receiving ANC and delivery care at Jacaranda Health from March 2016 to March 2018. Patients were selected using convenience and purposive sampling based on their risk status, parity, delivery date, and the services they received at Jacaranda Health. Eligible patients were identified through medical chart reviews and approached via a phone call made by a trained qualitative research assistant. Inclusion criteria consisted of: (1) pregnant women age 18 or over with a viable fetus attending ANC at Jacaranda Health in their first or second trimester and/or (2) women age 18 or over who delivered a live birth at Jacaranda Health. Risk status during pregnancy was categorized as low or high based on factors in a woman’s surgical, medical, and obstetric history or current pregnancy as specified in the facility’s high-risk protocol. Participants in the interviews and focus groups included a mix of high and low risk patients. Participants had attended ANC or delivered 0–4 months prior to the interview or focus group. Phone numbers for 135 patients meeting the eligibility criteria were identified through the Jacaranda Health patient database and called to seek consent for potential participation in the qualitative study. Of these, 68 (50%) potential participants were unreachable by phone after three attempts, and a further 17 (13%) potential participants were not able or not interested in participating. Several patients were able to combine their routine visits with the interview or focus group discussion. For those who declined participation, the most common reason was the inconvenience of returning to the hospital outside of a planned, routine visit for research purposes only. For some, the inconvenience was related to costs of getting to the facility, needing to find child care while they were out, or potential disapproval by their husband/partner.

During the implementation phase, patients participating in in-depth interviews (IDIs) and focus group dicussions (FGDs) had to meet additional eligibility criteria. Four months into the implementation phase, all eligible ANC patients had to have received a gestational dating ultrasound (between eight and 26 weeks of gestation) with the exception of three ANC patients who were interviewed about the reasons they opted out of the scan. Additionally, high-risk ANC patients were eligible if they fit the inclusion criteria above and had also undergone a fetal growth monitoring scan, had been referred internally due to their risk status, and/or had a newborn diagnosed as SGA. The eligibility criteria at 12 months mirrored that at four months except that all patients had to have delivered at Jacaranda Health. At this last data collection phase, two patients were also interviewed who opted out of the gestational dating scan and did not deliver at Jacaranda Health.

#### Data collection

Semi-structured guides containing questions and probes were used for the IDIs and FGDs. The guides were piloted prior to the start of data collection with five patients and adjusted for clarity based on feedback from the patients and interviewers. Qualitative data were collected via IDIs and FGDs from patients at three time points – once during the pre-implementation phase and twice during the implementation phase at four months and 12 months into the study. Data were collected at two time points in the implementation phase, eight months apart, to assess whether the stage of implementation of the standards into routine clinical practice made a difference in patient attitudes. Patients were given a small transportation stipend and refreshments in return for their participation. All participants were given the option to have the interview conducted in English or in Swahili. IDIs and FGDs were conducted by trained female research assistants (authors GK and TO) fluent in both English and Swahili. One interviewer held a masters degree (GK) and the other held a diploma (TO). Both had undergone human subjects research ethics courses and were employed by Jacaranda Health as research assistants during the period of the study. Each of the interviewers had 1–2 years of experience in leading interviews and focus groups with Jacaranda Health and 2 years at a different international research organization. Prior to data collection, the participants did not have contact with the interviewers except for a phone call to explain the study and arrange for the time of the interview. The participants were introduced to the interviewers as members of Jacaranda Health’s internal research team, and were assured that participation in the research would not impact service provision by the clinical team. The interviewers had no personal agenda to achieve through these interviews. The IDIs and FGDs were held in a private office area in a separate building within the hospital grounds, with no one else present besides the interviewer and note taker (FGDs only). IDIs lasted between 15–35 minutes and FGDs lasted between 30–60 minutes. The IDIs and FGDs were audio-recorded using a handheld audio recorder, uploaded to an encrypted database, and transcribed by a contracted, independent transcriber. The transcriber also translated the interviews to English (where applicable) and removed personally identifiable information before sharing transcripts with data analysts. The transcripts were not returned to the participants for additional feedback and repeat interviews were not conducted. Field notes were taken by the interviewers during IDIs and by a notetaker during FGDs, and were used by the research team to monitor progress and to identify potential emerging issues during data collection. Data saturation was monitored through reading of the transcripts by the analysis team and looking for the emergence of any unique viewpoints and themes. A summary of data collection methods can be found in Table 1. Written informed consent was obtained from all participants via signature.

**Table 1.** Qualitative data collection methods.

	Timeframe & Data Collection Mode					
	Pre-Implementation Phase		Implementation Phase 4-Months		Implementation Phase 12-Months	
Patient Classification	In-depth Interview	Focus Group Discussion	In-depth Interview	Focus Group Discussion	In-depth Interview	Focus Group Discussion
Low-risk	-	2 (7,4)a	3	1 (8)a	2	1 (4)a
High-risk	3	1 (3)a	4	-	7	-
Gestational Dating Opt-Outs	-	-	2	-	3	-
TOTAL	3	14	9	8	12	4
	**17**		**17**		**16**	

^a^Numbers in parentheses indicate the number of participants in the FGD

### Data analysis

Two experienced qualitative researchers analyzed and double-coded the transcripts using both *a priori* and inductive coding to identify themes using Nvivo 11 qualitative software [26]. A grounded theory approach was used to underpin the analysis. Double-coding was used to ensure reliability in the coding process and establish inter-coder agreement. Codes were developed in an iterative manner by the two researchers, with additional codes added through discussion of the data. Coding and themes were compared and agreed upon after each data collection round before continuing with further analyses. A thematic analysis was conducted by the researchers based on the coding, resulting in major and minor themes that reflected high-level topics from the guides and then narrowed to more specific groups of codes that had emerged from the data. Quotations that were illustrative of themes were also identified and are presented with the risk category and study time point in the results. Each round of data was analyzed independently and later compared after the final batch of data was analyzed. Demographic data for patients were obtained through Jacaranda Health’s Electronic Health Record Database and names were removed.

## Results

### Demographics

Fifty patients were interviewed during the study period; 17 (34%) were pregnant at the time of their interview and 33 (66%) had given birth within the last six months. The women who attended ANC and delivered at Jacaranda Health (28 patients) received, on average, three ANC visits at Jacaranda Health. Participants were, on average, 26 years old (range of 20–38 years), 45 (90%) were married, 37 (74%) were pregnant with or had recently given birth to their first child, and 36 (72%) had completed college or university. Of the fifty patients, 17 (34%) were designated as high-risk based on their clinical profile and clinical protocols. Demographic information can be found in Table 2.

**Table 2.** Demographics of participants.

Indicator of Interest	Pre-implementation	Implementation	
	Baseline N = 17	4-months N = 17	12-months N = 16
**Maternal age (years)** N Mean (SD) Range n (%) 20–29 30–34 35–39 40+ No data	17 25.9 (4.2) 22–35 14 (82%) 2 (12%) 1 (6%) 0 (0%) 0 (0%)	17 26.4 (3.8) 20–34 14 (82%) 3 (18%) 0 (0%) 0 (0%) 0 (0%)	16 28.3 (4.9) 21–38 9 (56%) 4 (25%) 2 (13%) 0 (0%) 1 (6%)
**Education** n (%) Primary Secondary College University or higher No data	0 (0%) 3 (18%) 7 (41%) 6 (35%) 1 (6%)	0 (0%) 5 (29%) 6 (35%) 5 (29%) 1 (6%)	0 (0%) 2 (13%) 9 (56%) 4 (25%) 1 (6%)
**Marital Status** n (%) Married/partnered Single No data	14 (82%) 2 (12%) 1 (6%)	15 (88%) 2 (12%) 0 (0%)	16 (100%) 0 (0%) 0 (0%)
**High risk status** n (%) Yes No data	6 (35%) 0 (0)	4 (24%) 0 (0)	7 (44%) 0 (0)
**Parity** n (%) 0 1 2–4 5+ No data	0 (0%) 14 (82%) 2 (12%) 0 (0%) 1 (6%)	6 (35%) 5 (29%) 6 (35%) 0 (0%) 0 (0%)	0 (0%) 12 (75%) 3 (19%) 0 (0%) 1 (6%)
**Any ANC attendance at JH** n (%) Yes No	17 (100) 0 (0)	17 (100) 0 (0)	12 (75) 4 (25)

The results that follow summarize patients’ descriptions of the facilitators and barriers to ultrasound uptake, as well as newborn size at birth assessment, including specific motivations noted by some of the women. A summary of facilitators and barriers to uptake and satisfaction can be found in Table 3.

**Table 3.** Summary of facilitators and barriers to patients’ uptake and satisfaction with ultrasound.

Facilitators	Barriers
Positive provider attitude and interactions Respect for authority of and advice from provider Assessment of well-being of fetus Knowledge of sex of fetus (when applicable) Well-informed estimate of date of delivery No cost for gestational dating scan Perceived accuracy of newborn size measurements High quality newborn equipment (scales, disposable head circumference tapes)	Wait times for gestational dating ultrasound Lack of awareness about gestational dating ultrasound Late ANC initiation Need for approval from husband/partner/family for ultrasound Poor communication with providers – lack of understanding regarding information provided by ultrasound, why recommended Knowledge that patient would not receive a printed picture of gestational dating scan Financial obstacles to multiple obstetric ultrasounds

### Ultrasound

#### Facilitators and barriers to uptake and satisfaction

Women were generally satisfied and spoke positively about their ultrasound experiences at Jacaranda Health. Most women reported having had two to three scans during their current pregnancy. Nearly all respondents (48 of 50 patients) felt that having one ultrasound was safe for the fetus and most would leave the number of ultrasounds to the discretion of a medical provider. Women most commonly cited the following functions of ultrasounds: checking the position of the fetus (15 of 29 patients), ensuring the general well-being of the fetus (12 of 29 patients), monitoring organ development and general growth of the fetus (8 of 29 patients), and checking the amniotic fluid (5 of 29 patients). In some cases, women also mentioned that the ultrasound allowed them to confirm their pregnancy, learn the estimated date of delivery (EDD), determine the sex of the fetus, and hear the fetus’ heartbeat.

“I can agree [to more scans] if it’s because of the doctor’s recommendation because she wants to check on the baby’s condition. I think it’s important I go by what the doctor is saying.” – Low Risk Patient (Pre-implementation Phase)

“I never know other reasons why [women get ultrasounds]. I only know that most people do the scans to know the gender of the baby but when I came here I knew that during the scan they can be able to detect if the baby has any problems and then they can be able to know the development of the kid if everything has formed and if there is any problem they will be able to know.” – Low Risk Patient (Implementation Phase – 4 months)

A major facilitator for ultrasound uptake in both study phases was reassurance of the health of the fetus (27 of 37 patients) through examination of its growth and position, and the ability to ‘see’ the fetus. The most frequently mentioned driver of ultrasound uptake was the desire to learn the sex of the fetus (32 of 37 patients). Other reported facilitating factors included the receipt of a printed picture of the scan (not possible for gestational dating ultrasounds on the device used), the gestational dating ultrasound being free, clear communication from providers regarding the rationale and benefits of the dating scan, and provision of an EDD in cases where a patient was unsure of her LMP.

“I wanted to confirm the delivery dates … the scan is very important to know how my baby is and it was also free so that helped me because I save something from my budget” – Low Risk Patient (Implementation Phase – 12 months)

All respondents reported that the EDD helped them to prepare for delivery financially and logistically (gather/source supplies, make child care arrangements, and organize transport). When patients were asked whether they would pay for the gestational dating scan if it were not free, many said they would. Both before and after the implementation of the INTERGROWTH-21^st^ standards, the most common barrier to receiving an ultrasound scan was a long wait time.

“My experience was good and I was being given good attention but the problem was the waiting time at the clinic” – High Risk Patient (Pre-implementation Phase)

A few patients expressed dissatisfaction due to their providers’ negative attitudes or lack of an adequate explanation of the procedure and results of an ultrasound. Based on patient reports, the information provided regarding the purpose, importance and results of the gestational dating ultrasound was not standardized. Most patients, at both timepoints, remembered being told broadly that the gestational dating scan is important for determining the well-being of the fetus. Patients specified that they wanted to be alerted about what to expect with the gestational dating scan well in advance of entering the nurse-midwife’s room, especially regarding the type of information they would and would not get from the scan. Patients also suggested producing and placing promotional materials with standardized messaging in visible areas around the clinic to increase awareness about the availability and purpose of the procedure, including the cost if a fee were to be charged.

“I would like [the ultrasound information] to be on the [TV] screen [in the reception area] because everyone passes through the receptionist area to wait. That would be better because at some point you will be able to see it.” – Low Risk Patient (Implementation Phase – 12 months)

Many patients expected a printed picture of the scan as proof of the procedure and/or knowledge of the sex of the fetus. Other cited barriers included paying for multiple ultrasounds, concerns about the safety of having multiple ultrasounds, confusion about what to expect from the ultrasound procedure, not ever learning that a free gestational dating scan was available, late initiation of ANC visits (22 of 39 patients first attended ANC during the second trimester), approval required from a husband/partner/family member, and lack of clarity about the purpose and cost of different types of obstetric scans. Amongst the patients who received ultrasounds (37 patients), only two said they were aware of the gestational dating scan before coming to Jacaranda Health. A small number of women reported being aware of myths and misconceptions in their communities that might hinder uptake of ultrasounds.

“These things the care providers write on the papers we cannot interpret them so unless you explain to me in a layman’s language I am not able to understand what is going on.” – High Risk Patient (Pre-implementation Phase)

The perception of accuracy of EDDs varied widely between individuals, regardless of whether the EDD was calculated by last menstrual period (LMP) or gestational dating scan. Women’s perception of accuracy of their EDD had to do with the proximity of the dates given (by scan or LMP) to their actual delivery date; the smaller the gap, the greater the perceived accuracy. Some women did not have a clear understanding that the EDD was an estimated date and rendered it inaccurate when they did not deliver on the exact date or within a few days of it. For several patients, nurse-midwives had given an EDD based on their LMP and then a separate date later based on their gestational dating scan. In some cases, the discrepancy between these EDDs calculated by LMP and the scan, or by different scans, led to dissatisfaction or confusion, and the assumption that one method was inaccurate.

### Newborn size at birth assessment

#### Facilitators and barriers to satisfaction

Women reported understanding that weight and length were common measurements taken at birth and were related to the health of the newborn. Patients who delivered at Jacaranda Health were generally satisfied with the perceived accuracy of newborn size at birth measurements. A few patients expressed that the scales and head circumference tapes used to measure their newborns seemed to be of higher quality than those used in other health facilities in which they delivered previously. Patients did not have as many comments regarding the newborn assessment as they did on other aspects of care (e.g. general antenatal and ultrasound care). However, some patients had reactions to the information they received about the size of their newborn at birth, which were facilitated or hindered by previous conversations with providers. For example, two women reported that fetal growth monitoring prepared them to anticipate that they would have smaller than average newborns. However, two other women were disappointed with the discrepancy between the predicted size shared during the antenatal scan and the actual birth weight, as both women were expecting to give birth to larger babies.

“I was just told the weight that was all … I was so tired … so long as the baby was healthy that was it.” – Low Risk Patient (Implementation Phase – 12 months)

“I expected a heavier baby. The last scan I did showed me that the weight was higher, but during birth the baby was actually smaller than I expected … I was slightly disappointed.” – High Risk Patient (Implementation Phase – 12 months)

## Discussion

This study aimed to examine women’s perceived facilitators and barriers to uptake, and attitudes and satisfaction with ultrasounds and newborn size at birth assessment before and during the implementation of the INTERGROWTH-21^st^ fetal and newborn growth standards. Two main themes emerged from the data: (1) the importance of understanding patients’ values and priorities to inform patient-centered care delivery and (2) aligning patient needs with clear provider communication and expectation-setting around ultrasound and newborn assessment services.

The most important factors women identified in their decision to choose ultrasounds were ensuring the well-being of their fetus and learning the sex. Understanding these priorities could allow a more tailored approach to communication around the gestational dating scan, both what it provides and cannot provide. For instance, learning the sex of the fetus was not always possible with the gestational dating scan due to the scan timing, which led to disappointment among some patients. The priorities of patients at Jacaranda Health regarding ultrasounds were similar to those identified by physicians in Rwanda [27]. Other studies have provided promising results about how women may value early ultrasound once they have experienced it. In a study conducted in Kilifi County in Kenya, all women who received an early ultrasound felt it had significant benefits, including reassurance of the fetus’s health and the ability to give health providers more information for accurate and timely diagnosis of potential complications, thus reinforcing its value as a protective measure during ANC [28]. Similar to other settings, wait times and cost remained important factors in women’s decisions to get ultrasounds [29].

When communication with patients was done effectively by providers, patients generally reported feeling comfortable and satisfied with the care they received using the new standards. Patients who received clear messaging from providers understood the value of obstetric ultrasounds and found the information a scan provided reassuring of the health of their pregnancy. In order to drive service utilization of gestational dating ultrasounds, women need to value the service being offered and understand its health benefits. Our findings suggest that this can be achieved through improvements in communication from providers, since women reportedly trust providers’ recommendations. Additionally, appropriate pricing, a clear understanding of the information that will be provided by the scan, and standardized communication materials were all cited as important factors in the successful implementation of the standards.

Interestingly, we did not observe any differences in patient perceptions between the four and 12 month data collection time points. We had hypothesized that as the tools became more integrated into routine clinical practice over time, patients might experience more standardized communication or have prior knowledge of the gestational dating scan when coming to the hospital. However, we did not observe such differences related to the duration of the implementation. The lack of observed differences may be related to the fact that the overall timeframe remained relatively short (12 months) and that patients could generally only reflect upon one personal experience with each of the INTERGROWTH-21^st^ tools, rather than comparing their experience with the same tools over time.

Previous studies in sub-Saharan Africa have also found that communication between providers and patients is a key factor in women’s satisfaction with care provision, specifically the uptake of ultrasounds [28, 30–32]. While most patients had a general understanding of the utility of ultrasounds, which they may have known before the scan or learned from the provider, the importance of the gestational dating scan was often unclear. Effective communication breaks down information in a way that is understandable to patients, regardless of the volume of information [33].

The newborn size at birth standards did not seem to elicit as much of a reaction from patients as the ultrasound required to implement the fetal growth standards. Patients were either not told or did not remember much information given about the size of their babies at birth beyond the birthweight and whether the baby was considered healthy for size. The procedures for newborn size assessment did not require changes in behavior, knowledge or attitudes of patients since measurements were taken in much the same way as during the pre-implementation period.

Our study was the first study evaluating the feasibility and acceptability of the INTERGROWTH-21^st^ fetal and newborn growth standards in a low-resource setting. A major strength of the study is that the implementation and evaluation included a specific focus on patient perspectives rather than making it secondary to other outcomes. Our study reinforces the fact that assessing the patient experience is an integral piece of implementation research, efforts to strengthen quality of care and establishment of sustainable interventions. Furthermore, the findings from our study provide a number of insights needed to prompt the demand side for ANC and ultrasound services. In particular, as the INTERGROWTH-21^st^ standards continue to be implementated around the world, it is important to ensure that patients recognize the value of the care they receive using the standards and are satisfied with it. Proactively seeking patients’ perspectives ensures that care is patient-centered.

Our study had several limitations. First, we were not able to interview the same patients at different data collection time points, as very few (if any) would have delivered in both 12-month study phases. However, in order to overcome this limitation, we made an effort to interview several patients who had given birth previously at Jacaranda Health. Second, our study focused on the experience of implementing the INTERGROWTH-21^st^ standards in a single, unique hospital. The patient reactions to the standards may differ in public hospitals, where the majority of low-income women seek care in Kenya. Finally, while the qualities of Jacaranda Health made it an ideal site to assess the implementation of the INTERGROWTH-21^st^ standards initially, the process would require adaptations when scaled up.

## Conclusion

The INTERGROWTH-21^st^ standards are prescriptive global standards now being utilized in several countries around the world for routine care, as well as in particular situations such as in the context of the Zika epidemic [34]. Critical information is gained by considering the perspective of patients when integrating new global standards into clinical practice. Increased and standardized communication about gestational dating scans should be provided before the ultrasound so that patients know what to expect. Communicating clearly will, in turn, lead to greater patient satisfaction and uptake of ultrasound services. The success of implementing gestational dating and fetal growth standards depends on women initiating ANC early enough to receive an accurate EDD, whether through ultrasound or other methods. Thus, implementers should consider investing in the development of community-based campaigns to encourage women to attend early ANC. Policy-makers should consider the promotion of early ultrasound where possible in conjunction with training providers on patient-centered care and communication. Considering the perspectives of patients will inform effective strategies for implementing the INTEGROWTH-21^st^ standards and other global growth standards, guidelines, and promising interventions in maternal and newborn care.

## Paper context

The INTERGROWTH-21^st^ fetal and newborn standards prescribe optimal growth in populations. Jacaranda Health in peri-urban Nairobi, Kenya was the first maternity in a low-resource setting to implement the standards. We qualitatively explored women’s experiences with the standards during ultrasound and newborn measurements. We contribute information on facilitators and barriers to implementing new standards and promoting early ultrasound uptake in low-resource maternal health contexts. Focus on standardized, clear communication from health providers is recommended.
